# Electrochemical Genosensing of *E. coli* Based on Padlock Probes and Rolling Circle Amplification

**DOI:** 10.3390/s21051749

**Published:** 2021-03-03

**Authors:** Alejandra Ben Aissa, Narayanan Madaboosi, Mats Nilsson, Maria Isabel Pividori

**Affiliations:** 1 Grup de Sensors i Biosensors, Departament de Química, Universitat Autònoma de Barcelona, 08193 Bellaterra, Spain; alejandra.benaissa@eurecat.org; 2 IIT Madras Bioincubator, Indian Institute of Technology, Chennai 600113, India; narayanan.srinivasan@scilifelab.se or; 3 Science for Life Laboratory, Department of Biochemistry and Biophysics, Stockholm University, 11419 Stockholm, Sweden; mats.nilsson@scilifelab.se; 4 Institute of Biotechnology and Biomedicine, Universitat Autònoma de Barcelona, 08193 Bellaterra, Spain

**Keywords:** isothermal amplification, rolling circle amplification, padlock probes, electrochemical genosensing, magnetic particles

## Abstract

Isothermal amplification techniques are emerging nowadays for the rapid and accurate detection of pathogenic bacteria in low resource settings, where many infectious diseases are endemic, and the lack of reliable power supply, trained personnel and specialized facilities pose critical barriers for timely diagnosis. This work addresses the detection of *E. coli* based on DNA isothermal amplification performed on magnetic particles (MPs) followed by electrochemical genosensing on disposable electrodes by square-wave voltammetry. In this approach, the bacterial DNA is preconcentrated using a target-specific magnetic probe and then amplified on the MPs by rolling circle amplification (RCA). Two different electrochemical readout methods for the RCA amplicons are tested. The first one relied on the labelling of the magnetic RCA product with a digoxigenin probe followed by the incubation with antiDIG-HRP antibody as electrochemical reporter. In the second case, the direct detection with an HRP-probe was performed. This latter strategy showed an improved analytical performance, while simultaneously avoiding the use of thermocyclers or bulky bench top equipment.

## 1. Introduction

*Escherichia coli* is the most common enteric Gram-negative bacteria. However, some strains, as extraintestinal pathogenic *E. coli* (ExPEC) [1] can cause illnesses in humans, including neonatal meningitis, sepsis, pneumonia, surgical site infections [2,3], and the urinary tract infections (UTIs) being the most common one [4]. These UTIs cause morbidity in all age groups and can be categorized according to the clinical syndrome, such as cystitis, pyelonephritis, catheter-related infection, or asymptomatic bacteriuria [5]. Importantly, UTIs caused by ExPEC represents a large burden in terms of health care costs [6], resulting in an estimated cost of billions of dollars annually [7]. Furthermore, an additional issue is related with the increasing antimicrobial resistance developed in the last years among the *E. coli* strains [8].

The incidence and the cost related to these infections would be reduced if reliable tests for the accurate and rapid diagnosis of the causative bacteria become widely available [9,10]. Conventional microbiological techniques are currently the gold standard for identification of pathogenic bacteria, although they are time-consuming [11]. Common approaches for the rapid detection of bacteria usually include immunoassays and nucleic-acid amplification tests (NAATs). The first generation of NAATs based on PCR is not amenable with rapid diagnostic tests (RDTs), since it requires thermocycling platforms, trainee personnel and infrastructure including reliable power supply, which constitute a critical barrier for PCR in resource-limited settings (RLS) [12]. Recent work on implementing RDTs in RLS has focused on portable platforms operated by batteries [13], with smartphone-based diagnostics widening their scope [14,15]. Many companies have launched portable PCR platforms that can be operated with batteries, including Palm PCR™ (Ahram Biosystems Inc.), Freedom4 (Ubiquitome), miniPCR (Amplyus), among others, that are commercially available. Another approach involves isothermal amplification techniques that do not require temperature cycling [16]. These methods use a variety of reaction principles to specifically amplify nucleic acid targets through isothermal conditions, and the amplicons can be detected even without the need for any instrument. Important advances in the integration of Rolling Circle Amplification (RCA) in an ultrasensitive electrochemical genosensor were recently achieved and described by our research group [17] and have also been demonstrated using clinical samples [18]. This technique is based on the endlessness of a circle [19]. The circle DNA complementary to the target is formed using a padlock probe (PLP), by circularization with a highly specific enzymatic ligation, which is followed by an amplification using DNA polymerase. Upon amplification, long DNA repeats that get formed collapse into a condensed blob-like structure, which can then be readily and sensitively detected by hybridization with a tagging probe. Importantly, RCA has no requirement of special instrumentation to cycle the temperature, contrary to PCR-based DNA diagnostics.

This work addresses the detection of *E. coli* based on DNA isothermal amplification [20] using PLPs and electrochemical genosensing on commercial screen-printed electrodes by square-wave voltammetry, with outstanding sensitivity. Moreover, this approach can be integrated with magnetic separation for a further increase of the sensitivity, by using an oligonucleotide coupled to magnetic particles (MPs) [21]. The integration of magnetic particles to the assay allows the use of high concentration of PLPs, which reduces the assay and ligation time from hours to several minutes [22,23]. Once the ligation is finished, the unreacted PLPs are easily removed by magnetic actuation. Progress in this area will, without any doubt, have direct impact in the detection of communicable diseases, since even with the best current technologies, amplification of the genetic material for certain applications cannot be avoided.

## 2. Materials and Methods

### 2.1. Instrumentation

The electrochemical measurements were performed on carbon screen-printed electrodes (ref. DRP-110) using a portable bipotentiostat DRP-STAT200 operated by DropView 2.2 for instrument control and data acquisition (Metrohm DropSens, Oviedo, Spain). Fluorescently labelled rolling circle products were detected using Aquila 400 (Q-linea AB, Sweden).

### 2.2. Chemicals and Biochemicals

Streptavidin magnetic particles (MPs) (Dynabeads^®^ MyOne Streptavidin T1 Prod. No. 65601) were purchased from Life Technologies. Synthetic DNA, padlock probe and Cy3 labelled detection probes were produced by Integrated DNA technologies (Belgium), and their sequences described in Table 1. Peroxidase-modified probes were provided by Biomers, Germany. All other solutions were prepared with Milli-Q water and other reagents were in analytical reagent grade (supplied from Sigma and Merck), as described in Supplementary Materials. The composition of these solutions was: (i) phosphate buffer for electrochemical measurement (ePBS): 0.1 mol L^−1^ Na_2_HPO_4_, 0.1 mol L^−1^ NaCl, (ii) Hybridization buffer: 6.6 mmol L^−1^ Tris-acetate pH 7.9, 2 mmol L^−1^ magnesium acetate, 13 mmol L ^−1^ potassium acetate, 0.02% Tween-20, 0.2 mmol L ^−1^ DTT and (iii) washing buffer: 10 mmol L^−1^ Tris-HCl pH 7.5, 5 mmol L^−1^ EDTA, 0.1% Tween-20 and 0.1 mol L^−1^ NaCl.

### 2.3. Design of the Padlock, Capture and Readout Probes

**Figure 1:**
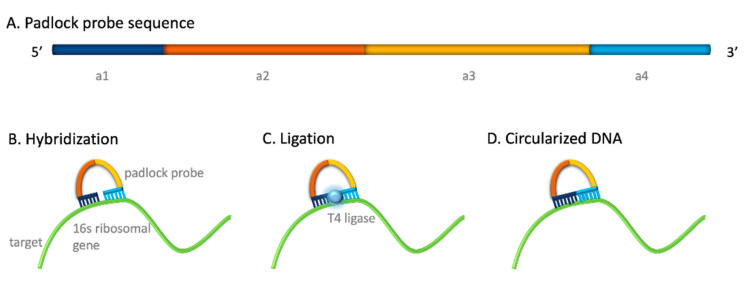
(**A**) Design of the specific padlock probe for the detection of *E. coli* comprising two complementary sequences to the target (a1 and a4) and two sequences (a2 and a3) to achieve the readout. (**B**) Hybridization of the padlock probe to the 16S ribosomal *E. coli* gene. (**C**) Ligation by the T4 DNA ligase enzyme. (**D**) Circularized DNA templates obtained after ligation of the padlock probes and used for the rolling circle amplification.

A PLP is a linear, 80–90 bases long, oligonucleotide that consists of at least three aligned segments: two complementary sequences located at the 5′ and 3′ ends which hybridize the DNA target, and a central non-complementary region, which hybridizes the readout probes. Specifically, as shown in Figure 1A, the two complementary sequences located at the 5′ and 3′ ends are indicated as a1 (17 bp) and a4 (18 bp). Further details can be found in Supplementary Materials, including Figure S1 and Table S1, in which each sequence is highlighted. The selected DNA target sequence is specific for *Escherichia coli* 16S ribosomal gene (Genbank accession Nº CP016182.2). The 16S ribosomal gene from *E. coli* was selected as this gene is directly involved in various events of the central dogma (including discrimination of mRNA initiation sites, tRNA binding and association of the two ribosomal units) [24]^.^ This gene is comprised of highly conserved regions flanking hypervariable regions [25]. By blasting the selected sequence, high specificity towards *E. coli* is found in different isolates, including 100% homology from different sources, ranged from environmental samples isolates (GenBank: LR738970.1), clinical isolates (GenBank: CP062970.1), food safety (GenBank: AP023286.1), among many others. Besides, the high copy number repetitions of this sequence reported (GenBank CP058342.1, AP023286.1, CP047710.1) envisages an increased the sensitivity for the detection of the whole bacteria.

After hybridization of both ends of the PLP to the DNA target sequence for *E. coli* (Figure 1B) and further ligation (Figure 1C), the linear probe is then converted to a DNA circle (Figure 1D).

The PLP also comprises two sequences located in the center indicated as a2 (14 b) and a3 (35 bp), in Figure 1A, non-complementary to the target but which hybridize with the readout probes, modified with different tags for indirect (digoxigenin) and direct (Cy3 and HRP) labelling and to achieve fluorescence and electrochemical readouts. More details can be also observed in in Supplementary Materials, including Figure S1 and Table S1.

In order to achieve the target isolation and preconcentration based on magnetic actuation, a biotinylated capture probe, was designed to hybridize a specific sequence located upstream of the *E. coli* recognition sequence (detailed in Figure S1 and Table S1). Thus, streptavidin-magnetic particles can bind to the biotinylated probe and preconcentrate the sample. Finally, synthetic target for *Escherichia coli* 16s rRNA was designed. Table 1 summarizes the oligonucleotide sequences used in this study. Further details can be found in Table S1, in which the different sequences involved in hybridization and readout are highlighted.

### 2.4. Selection of the Readout Probe Sequence and Optimization of the Rolling Circle Amplification

**Figure 2:**
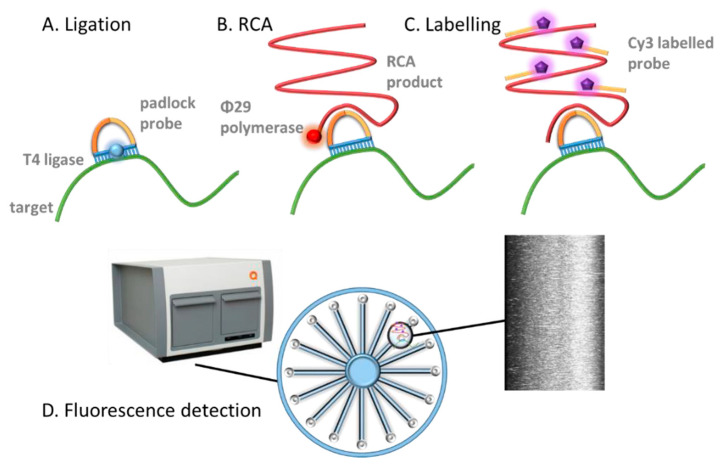
Schematic representation of the rolling circle amplification readout based on fluorescence. (**A**) Padlock probe is hybridized on the bacterial DNA target, followed by ligation; (**B**) Rolling circle amplification (**C**) Hybridization with the Cy3 readout probe acting as fluorescent reporter; (**D**) Detection with Aquila 400 detection equipment.

Different parameters were firstly evaluated for the RCA based on the fluorescent readout (schematically shown in Figure 2).

To achieve that, a dilution series of 16S ribosomal *E. coli* synthetic target was performed from 1 fM to 1 pM. PLP ligation mix was added to each sample, comprising 10 nmol·L^−1^ padlock probe, 0.2 mg mL^−1^ BSA, 0.68 mmol L^−1^ ATP, 1x T4 ligase reaction buffer and 5 U T4 ligase. The reaction was incubated at 37 °C for 15 min. After that, RCA mix containing 1x phi29 DNA polymerase buffer, 125 μM dNTPs, 0.2 mg ml ^−1^ BSA and 6 U phi29 DNA polymerase was added to the circularized DNA and RCA was performed at 37 °C for 60 min. RCPs cannot be resolved by gel electrophoresis due to the broad smear of the high molecular weight DNAs. In all instances, in order to achieve the fluorescence readout, RCPs were fluorescently labelled by using readout probe 3 or 4 (Table 1), labelled with the fluorescence tag Cy5. Aquila 400 amplified single molecule counter (Q-linea, Uppsala) was used to record the fluorescence readout and to count the RCA products. Finally, 20 µL of the total sample was analyzed using the Aquila 400 amplified single molecule counter. This method was used to achieve the optimal conditions, including the *composition of hybridization buffer, labelling temperature*, *readout probe sequence and concentration*, as described below:

*Hybridization buffer.* Four hybridization buffers were studied for the labelling process, being their compositions.

*hybridization buffer A.* 6.6 mmol L^−1^ Tris-acetate pH 7.9, 2 mmol L^−1^ magnesium acetate, 13 mmol L ^−1^ potassium acetate, 0.02% Tween-20, 0.2 mmol L ^−1^ DTT. *hybridization buffer B.* 20 mmol L^−1^ EDTA, 40 mmol L^−1^ Tris-HCl, 2.8 mol L^−1^ NaCl, and 0.2% Tween-20.

*hybridization buffer C.* 20 mmol L^−1^ EDTA, 40 mmol L^−1^ Tris-HCl, 2.8 mol L^−1^ NaCl, 0.2% Tween-20, 30 mM trisodium citrate and 40% formamide.

*hybridization buffer D.* 40 mM Tris-HCl pH 8.3, 50 mM KCl, 20 mM MgCl_2_, 1 mM NAD, 0.02% Triton^®^ X-100.

*Labelling temperature optimization.* A binding step at high temperatures is usually included during the hybridization process due to its demonstrated improvement in terms of time. In order to evaluate the effect on the yield of the labelling, 20 µL of RCP were incubated in hybridization buffer with 25 nmol L^−1^ of readout probe 4. In procedure 1, the mix was incubated at 75 °C for 2 min, after that another incubation at 37 °C for 45 min was performed. The procedure 2 consisted in one only incubation at 37 °C for 45 min.

*Readout probe sequence.* A comparison of two different readout probes (as shown in Table 1) labelled in 5′with Cy3 was performed in order to achieve the direct fluorescence readout.

Readout probe 3 CTTGCGACGTCAGTGGATAGTGTCTTACACGATTT

Readout probe 4 AGAGTGTACCGACCTC

*Readout probe concentration.* Concentrations ranging from 10 to 25 nmol L^−1^ of the readout probe were studied in order to evaluate its influence during the labelling process.

### 2.5. Rolling Circle Amplification on Streptavidin Magnetic Particles and Electrochemical Genosensing

**Figure 3:**
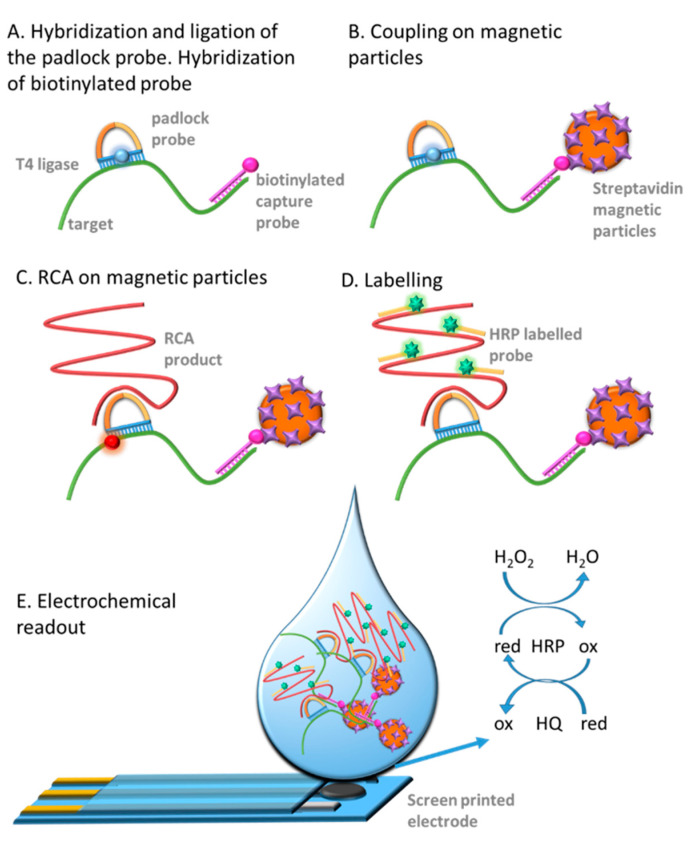
Schematic representation of the electrochemical genosensing for the rolling circle amplification products detection on streptavidin-MP. (**A**) The biotinylated capture probe as well as the padlock probe are both hybridized to the DNA target (**B**) The DNA target is then coupled on streptavidin magnetic particles; (**C**) RCA on the streptavidin-MP is then performed; (**D**) Hybridization with the readout probe (HRP labelled) to achieve the electrochemical reporter is then performed; (**E**) Electrochemical readout by square wave voltammetry (SWV) upon addition of the mediator HQ and the substrate H_2_O_2_ for the HRP on screen-printed electrodes.

The procedure is schematically described in Figure 3 and detailed described in Supplementary Materials, involving the following steps, as previously described by our research group [17]: (A) Hybridization and Ligation. Each sample of DNA target was incubated with the biotinylated capture probe, in order to achieve the preconcentration of the RCPs on streptavidin magnetic particles in (B) Coupling on streptavidin magnetic particles, followed by (C) Rolling Circle amplification and (D) Labelling with the readout probe labelled with HRP. After each incubation or washing step, a magnetic separator was positioned under the tubes until pellet formation occurred on the tube’s side wall, followed by supernatant separation.

Finally, (E) electrochemical readout of RCPs attached on the streptavidin-MPs was performed on carbon screen-printed electrodes with a portable bipotentiostat connected by a universal USB port to a laptop computer operated by batteries (as detailed described in Supplementary Materials and Figures S3 Figure S4 therein), by square wave voltammetry (SWV) measurements. The maximal signal obtained in the cathodic peaks was used for the electrochemical signal plotted in the results.

### 2.6. Direct and Indirect Readout Approach

In order to optimize the electrochemical detection of RCPs, two different electrochemical readout methods for the RCA amplicons on MPs were tested. The first one relied on the indirect labelling of the magnetic RCA product with a digoxigenin probe (readout probe 2, Table 1) followed by the incubation with antiDIG-HRP antibody as electrochemical reporter. In the second case, the direct detection with an HRP-probe (readout probe 1, Table 1) was performed. In both instances, 16S ribosomal *E. coli* synthetic target (1 nM) was incubated for 20 min at 55 °C with 50 nmol L^−1^ of biotinylated capture oligo in PLP ligation mix. In the direct labelling, this procedure was performed by the incubation with the readout probe 1 labelled with HRP (20 nmol L^−1^) in hybridization buffer A at 37 °C for 45 min. The indirect labelling was performed by the incubation with the readout probe 2 labelled with digoxigenin (20 nmol L^−1^) in the same hybridization buffer A at 37 °C for 45 min. After that, to come up with the indirect label, a further incubation step was performed with antiDIG-HRP antibody in PBS buffer with 1% BSA at 37 °C for 30 min. In both instances, labelled RCA products were washed (x3) to remove the unbound reagents. Negative controls, performed with all the components with the exception of the template bacterial DNA, were in all instances prepared.

After each incubation or washing step, a magnetic separator was positioned under the tubes until pellet formation on the tube’s side wall, followed by supernatant separation.

### 2.7. Electrochemical Genosensing of E. coli.

The electrochemical genosensor was evaluated for the detection of *Escherichia coli*. For that, rolling circle amplified DNA coming from an overnight culture of *E. coli* DH5α were evaluated. The bacteria *E. coli* was routinely grown in sterile liquid Luria Bertani (LB) broth for 18 h at 37 °C under aerobic conditions. After that, serial dilutions from the culture were performed and 100 µL of each dilution was spiked on LB agar plates. After incubating the plate at 37 °C for 24 h, the culture colonies on the plates were counted to estimate the number of viable bacteria in CFU mL^−1^. On the other hand, cells from 1 mL of each sample were lysed and the DNA was extracted.

For the DNA extraction, 1 mL of each sample were centrifuged, and the cell pellets were resuspended in 1 mL of sterile water. The resuspended cells were re-centrifuged at 12,500 g for 15 min, after that, the cells were resuspended in 50 µL of Tris–EDTA buffer and then kept in a boiling water bath for 10 min. Immediately after heat denaturation, the samples are cooled on ice for 5 min. The hybridization of the capture probe and the ligation was immediately performed by incubating the *E coli* template (10 µL of the extracted *E coli* DNA) at the same time with the padlock and capture probe, which was after coupled to 4 μL of streptavidin-magnetic particles for 5 min at RT. Then, the sample-bead complex was washed in order to eliminate the padlock excess and to remove the bacterial matrix and resuspended in RCA mix for the isothermal amplification. RCA was performed for 60 min at 37 °C. A negative control without *E coli* DNA, which had the same treatment as the positive sample, was processed. The electrochemical genosensing of the RCPs was performed by the hybridization with the readout probe 1 (Table 1) labelled with HRP (20 nmol L^−1^) (as schematized in Figure 3) in hybridization buffer A for 45 min at 37 °C, followed by washing steps under magnetic actuation, to eliminate the excess of readout probe.

## 3. Results and Discussion

### 3.1. Selection of the Readout Probe Sequence and Optimization of the Rolling Circle Amplification

**Figure 4:**
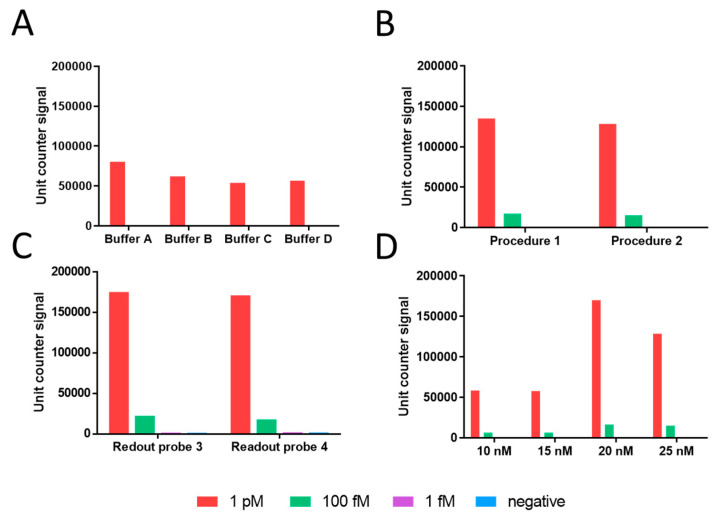
Results for the optimization of the rolling circle amplification. The results are shown as bars including the Unit counter signal. (**A**). Hybridization buffer optimization. (**B**). Labelling temperature optimization (Procedure 1: incubation at 75 °C for 2 min + 37 °C for 45 min; Procedure 2: incubation at 37 °C for 45 min); (**C**). Readout probe sequence. (**D**): Readout probe optimization. Further experimental details can be found in the main text. In all instances, the negative control without the DNA template, were performed (blue bar).

Different parameters were firstly evaluated for the RCA based on fluorescence readout and using Aquila 400 (Q-linea AB, Sweden). The results, shown in Figure 4, include the optimized conditions, such as the *composition of hybridization buffer* (Figure 4A), *labelling temperature* (Figure 4B), *readout probe sequence* (Figure 4C) *and concentration* (Figure 4D).

To summarize, Buffer A exhibits improved results for labelling at 37 °C for 45 min with 20 nmol L^−1^ of readout probe with sequence as 3 (the same as sequence 1 and 2, with different tags) (Table 1). Based on the obtained results, all further studies for the RCA on streptavidin magnetic particles and electrochemical genosensing were performed by using these optimized conditions.

### 3.2. Rolling Circle Amplification on Streptavidin Magnetic Particles and Electrochemical Genosensing. Direct and Indirect Readout Approach

**Figure 5:**
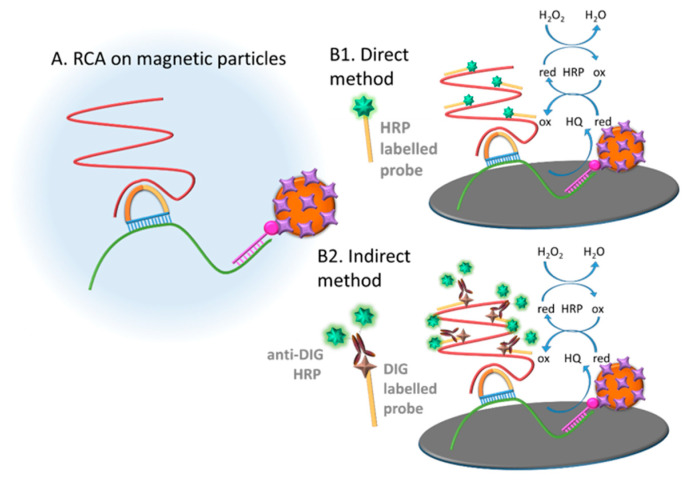
Schematic representation of the electrochemical genosensing of E. coli by rolling circle amplification. (**A**) RCA on the streptavidin-MPs; (**B1**) Direct labelling. Hybridization with the readout probe 1 (HRP labelled) acting as electrochemical reporter. (**B2**) Indirect labelling. Hybridization with the readout probe 2 (digoxigenin modified) and incubation with the electrochemical reporter antiDIG-HRP. In both cases, the electrochemical determination is performed by square wave voltammetry upon addition of the mediator HQ and the substrate H_2_O_2_ for the HRP.

Figure 5 shows schematically the different approaches (direct and indirect) for the labelling of the rolling circle amplification products (RCPs) for the subsequent electrochemical readout.

**Figure 6:**
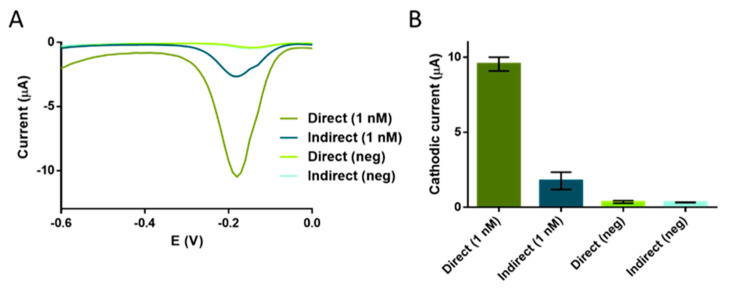
Comparative study on direct and indirect readout systems for the rolling circle amplification and electrochemical genosensing. For both systems, 1 nM of circularized bacterial DNA were used, except for the negative controls. (**A**) Raw square wave voltammetry data. (**B**) Graph bars of the SWV maximal signal. The bars represent the mean value of the maximal signal and the error bars, the standard deviation for n = 3. Medium: phosphate buffer. Mediator: hydroquinone 1 mmol L^−1^. Substrate: H_2_O_2_ 0.25 mmol L^−1^. The potential range was 0.1 to −0.7 V with potential step and amplitude of 10 mV and frequency of 1 Hz. For both systems, the negative control without the DNA template, were performed (n = 3).

The direct labelling consists of the hybridization of the magnetic RCP (obtained by synthesizing the RCP on a MP) with a readout probe directly modified with the bulky HRP (readout probe 1). The second approach relied on the indirect labelling with a digoxigenin probe (readout probe 2, Table 1) followed by the incubation with antiDIG-HRP antibody as electrochemical reporter. In both instances, the RCPs on the MPs were then electrochemically detected. The results are shown in Figure 6.

Although a higher hybridization rate was expected for the readout probe 2 based on digoxigenin tag and the indirect approach due to the smaller size, it seems to be hindered by the subsequent recognition of the antibody, thereby providing poorer performance than the direct labelling with the readout probe 1. For the concentration of 1 nM (10 fmol) of 16S ribosomal *E. coli* synthetic target, a mean value of 9.52 μA/SD 0.4 μA (direct labelling) and 1.76 μA/SD 0.5 μA (indirect labelling) were obtained for the two methods. The mean values for the background signals were 0.32 μA/SD 0.02 μA (for direct labelling), 0.42 μA/SD 0.09 μA (for indirect labelling). The signal-to-background ratios were 29.75 and 4.19 for direct and indirect labelling, respectively, confirming an increased performance of the direct labelling for the detection of the RCPs. It is important to highlight that the RCPs were previously described as a blob of DNA [26] in which some of the repeated hybridization sequences might be in the interior, and thus being less accessible for further reactions. This direct readout system was used in further experiments.

### 3.3. Rolling Circle Amplification on Streptavidin Magnetic Particles and Electrochemical Genosensing

**Figure 7:**
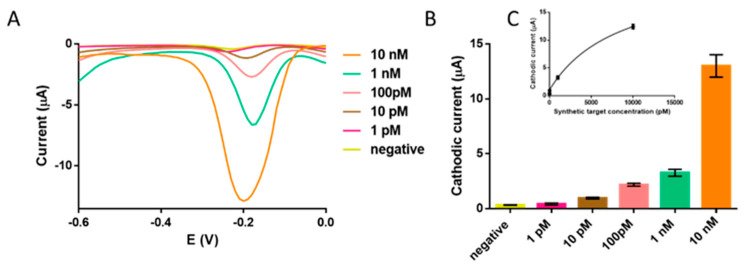
(**A**) Voltagramms for the RCA on MPs and electrochemical genosensing based on direct labelling, performed by isothermal amplification of 16S ribosomal *E. coli* synthetic target ranging from 1pM to 10 nM, and including the negative controls. All other conditions as Figure 6. (**B**) Calibration plot of the SWV maximal signal. The bars represent the mean value, and the error bars, the SD for n = 3. (**C**) Fitted SWV signals by a nonlinear regression, nonlinear regression (Two site binding –hyperbola). The negative controls are also shown (n = 6).

To determine the performance and limit of detection (LOD) of the RCA on streptavidin-MPs, the electrochemical genosensing was performed on a serial dilution of 16s ribosomal *E. coli* synthetic target, ranging from 1 pM to 10 nM (10 amol to 0.1 pmol), and detected by square wave voltammetry. The results are shown in Figure 7.

The maximal signals obtained in the voltagramms are presented in Figure 7A. The LOD was estimated by fitting the raw data using a nonlinear regression (Two site binding –hyperbola) (R^2^ = 0.9965), as shown in Figure 7C, by processing the negative control samples (n = 6) obtaining a mean value of 0.32 μA with a standard deviation (SD) of 0.02. The cut-off value was then determined with a one-tailed t test at a 95% confidence level (*t* = 2.015), giving a value of 0.36 μA. The LOD was found to be 6.7 amol (4,03 × 10^6^ DNA copies) in 10 μL of sample (0.67 pM, corresponding to 4.03 × 10^11^ DNA copies L^−1^).

It is important to highlight that the limit of detection reached in this work with the electrochemical detection is similar or even improved than other reported bacterial detection methods where RCA had been used for the signal amplification [27] (Table S2, Supplementary Materials, and references therein). Further improvement of the LOD of *E. coli* at clinically relevant levels, can be achieved by re-amplification of the RCA products by C2CA [17].

### 3.4. Electrochemical Genosensing of E. coli

**Figure 8:**
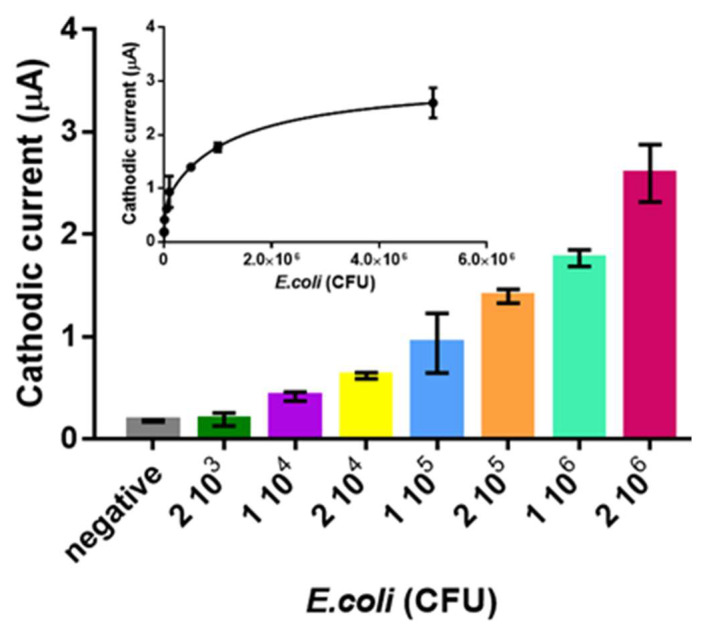
Rolling circle amplification and electrochemical genosensing of *E. coli* samples. The plots show the SWV maximal signal. The bars represent the mean value of the maximal signal and the error bars, the standard deviation for n = 3. All other conditions as Figure 6, including the negative controls are also shown (n = 6).

Finally, the electrochemical genosensor was evaluated for the detection of *E. coli*. The samples were treated according to the experimental section. Figure 8 shows the maximum signal obtained in the voltagramms for the *E coli*. The LOD was found to be 3 × 10^3^ CFU in 10 µL of sample, corresponding to 10^4.3^ CFU mL^−1^.

It was estimated as above, by fitting the raw data using a nonlinear regression (Two site binding –hyperbola) (R^2^ = 0.9513). Regarding the stability, it is determined by the stability of the reagents (oligonucleotides, strepAv-MP, RCA mix and enzymes), that were kept at 4 °C as recommended by the manufacturers. As the screen-printed electrodes are not biologically modified, they were storage at RT, as recommended by the manufacturer, and use them within the expiration date.

According to these results, it was demonstrated that the system presented in this work, combining the electrochemical genosensing strategy with RCA, offers a sensitive method for the bacterial detection and promising features to be used in Low resource settings, when compared with other methods (Table S2 and references therein, Supplementary Materials).

## 4. Conclusions

The design of low cost, user friendly and point of care devices able to provide rapid results with high sensitivity and specificity is nowadays a major challenge. With recent advances in molecular methods, the combination of NAAT using isothermal assays with electrochemical sensor is a highly suitable approach to reach this objective. This work reports an electrochemical genosensor for the detection of bacterial DNA by using PLPs and the subsequent amplification with RCA. This strategy is a powerful combination for highly specific and sensitive *E. coli* DNA detection that can be applied in RLS. The incorporation of MPs in the assay brings several advantages besides the preconcentration under magnetic actuation, including their large surface to volume ratio, facilitating high capture efficiencies and reaction kinetics. Other important advantages include the ease of washing procedure, simplified assay steps and reduced assay time. The specificity provided by the ligation of PLPs and the isothermal nature of the RCA make it an attractive method for the application at the practitioner site. Moreover, the use of electrochemical readout offers a robust test that can be performed with hand-held equipment operated with batteries. This approach requires minimal training for final user and provide rapid results for taking action immediately, for example prescribing without any dilation the treatment with antibiotics at first visit.

## Figures and Tables

**Table 1:** Oligonucleotide sequences used in this study.

Description	Sequence	Label
Padlock probe	[Phos]GTTACCCGCAGAAGAAGAGTGTACCGACCTCAGTATCTTGCGACGTCAGTGGATAGTGTCTTACACGATTTATACCTTTGCTCATTGAC	none
16s ribosomal *E.coli* synthetic target	TAACGCTTGCACCCTCCGTATTACCGCGGCTGCTGGCACGGAGTTAGCCGGTGCTTCTTCTGCGGGTAACGTCAATGAGCAAAGGTATTAACTTTACTCCCTTCC	none
Capture probe	CTCTCTCTCTCGTGCCAGCAGCCGCGGTAATACGGAGGGTGCAAGCGTTA	5′ Biotin (magnetic separation)
Readout probe 1	CTTGCGACGTCAGTGGATAGTGTCTTACACGATTT	5′ HRP (direct electrochemical readout)
Readout probe 2	CTTGCGACGTCAGTGGATAGTGTCTTACACGATTT	5′ DIG (indirect readout)
Readout probe 3	CTTGCGACGTCAGTGGATAGTGTCTTACACGATTT	5′ Cy3 (direct fluorescence readout)
Readout probe 4	AGAGTGTACCGACCTC	5′ Cy3 (direct fluorescence readout)

## Data Availability

Not applicable.

## Supplementary Materials

The following are available online at https://www.mdpi.com/1424-8220/21/5/1749/s1. Table S1. Oligonucleotide sequences used in this study. In the padlock probe, the sequences are tagged in blue (dark a1 and light a4), corresponding to the regions complementary to the DNA target, while orange (a2) and yellow (a3) regions correspond to the readout probes. Pink shows the capture oligonucleotide region complementary to the target. The different complementary regions have been underlined in the same way. Table S2. Analytical performance and main characteristics of other related biosensors or platforms based on isothermal amplification for the detection of E. coli or other bacteria. Figure S1. Design of the specific padlock probe for the detection of E. coli comprising two complementary sequences to the target (a1 and a4) and two sequences (a2 and a3) to achieve the readout. The schematic location of the capture probe is also shown. Figure S2. Schematic representation of the electrochemical genosensing for the rolling circle amplification products detection on streptavidin-MP. (A) The biotinylated capture probe as well as the padlock probe are both hybridized to the DNA target (B) The DNA target is then coupled on streptavidin magnetic particles; (C) RCA on the streptavidin-MP is then performed; (D) Hybridization with the readout probe (HRP labelled) to achieve the electrochemical reporter is then performed; (E) Electrochemical readout by square wave voltammetry upon addition of the mediator HQ and the substrate H2O2 for the HRP on screen-printed electrodes. Figure S3. Configuration of the commercial screen-printed electrodes used in this work and image of the Boxed Connector for Screen-Printed Electrodes. It can be appreciated the drop on the Surface of the working carbon electrode (images obtained from Dropsens). Figure S4. Enzymatic mechanism of the HRP enzyme conjugated to the readout probes on the surface of the carbon screen-printed electrode, upon addition of H2O2 as a substrate and hydroquinone (HQ) as a mediator.
