# Supplementary material for: Electrochemical Genosensing of E. coli Based on Padlock Probes and Rolling Circle Amplification
Source: Sensors (Basel). 2021 Mar 3;21(5):1749. doi: 10.3390/s21051749 (PMC7959471; doi:10.3390/s21051749)
Supplement: Supplementary file 1 [file sensors-21-01749-s001.pdf]

sensors
MDPI

Supplementary Material

Electrochemical Genosensing of E. coli Based on Padlock Probes and Rolling Circle Amplification

Alejandra Ben Aissa [1], Narayanan Madaboosi [2,3], Mats Nilsson[3] and Maria Isabel Pividori [1,4,*]

[1] Grup de Sensors i Biosensors, Departament de Química, Universitat Autònoma de Barcelona, 08193 Bellaterra, Spain; alejandra.benaissa@eurecat.org
[2] IIT Madras Bioincubator, Indian Institute of Technology, Chennai 600113, India; narayanan.srinivasan@scil-ifelab.se, narayanan@bioincubator.iitm.ac.in
[3] Department of Biochemistry and Biophysics, Science for Life Laboratory, Stockholm University, 114 19 Stockholm, Sweden; mats.nilsson@scilifelab.se
[4] Institute of Biotechnology and Biomedicine, Universitat Autònoma de Barcelona, 08193 Bellaterra, Spain.
* Correspondence: Isabel.Pividori@uab.cat; Tel.: +34-93-581-1976

2. Materials and Methods

2.1. Instrumentation

The electrochemical measurements were performed on carbon screen-printed electrodes (ref. DRP-110) using a portable bipotentiostat DRP-STAT200 operated by DropView 2.2 for instrument control and data acquisition (Dropsens, Spain). Fluorescently labelled rolling circle products were detected using Aquila 400 (Q-linea AB, Sweden).

2.2. Chemicals and biochemicals

Streptavidin magnetic particles (MPs) (Dynabeads® MyOne Streptavidin T1 Prod. No. 65601) were purchased from Life Technologies. Synthetic DNA, padlock probe and Cy3 labelled detection probes were produced by Integrated DNA technologies (Belgium). Peroxidase-modified probes were provided by Biomers, Germany. T4 ligase with its corresponding reaction buffer (66 mmol L^{-1} Tris-HCl pH 7.5, 10 mmol L^{-1} DTT, 10 mmol L^{-1} $MgCl_2$) was purchased from Blirt (Gdansk, Poland). Phi29 DNA polymerase as well as its corresponding buffer (33 mmol L^{-1} Tris-acetate pH 7.9, 10 mmol L^{-1} magnesium acetate, 66 mmol L^{-1} potassium acetate, 0.1% Tween-20, 1 mmol L^{-1} DTT) were from Monserate, USA. T4 ligase reaction buffer (66 mmol L-1 Tris-HCl pH 7.5, 10 mmol L-1 DTT, 10 mmol L-1 MgCl2) was purchased from DNA Gdansk. All solutions were prepared with Milli-Q water and other reagents were in analytical reagent grade (supplied from Sigma and Merck). The composition of these solutions were: i) phosphate buffer for electrochemical measurement (ePBS): 0.1 mol L^{-1} Na_2HPO_4, 0.1 mol L^{-1} NaCl, ii) Hybridization buffer: 6.6 mmol L^{-1} Tris-acetate pH 7.9, 2 mmol L^{-1} magnesium acetate, 13 mmol L^{-1} potassium acetate, 0.02% Tween-20, 0.2 mmol L^{-1} DTT and iii) washing buffer: 10 mmol L^{-1} Tris-HCl pH 7.5, 5 mmol L^{-1} EDTA, 0.1% Tween-20 and 0.1 mol L^{-1} NaCl。

Table S1. Oligonucleotide sequences used in this study. In the padlock probe, the sequences are tagged in blue (dark a1 and light a4), corresponding to the regions complementary to the DNA target, while orange (a2) and **yellow** (**a3)** regions correspond to the readout probes. Pink shows the capture oligonucleotide region complementary to the target. The different complementary regions have been underlined in the same way.

Description	Sequence	Label
Padlock probe	[Phos]GTTACCCGCAGAA-GAAGAGTGTACCGAC-CTCAGTATAT**CTTGCGAC-GTCAGTGGA-TAGTGTCTTACACGATT T**ATACCTTTGCTCATT-GAC	none
16s ribosomal E.coli synthetic target	TAACGCTTGCACCCTCCG-TATTACCGCGGCTGCTGG-CACGGAG-TTAGCCGGTGCTTCTTCTG CGGGTAACGTCAATGAG-CAAAGGTATTAACTTTAC TCCCTTCC	none
Capture probe	CTCTCTCTCTCGTGCCAG-CAGCCGCGGTAATAC-GGAGGGTGCAAGCGTTA	5′ Biotin (magnetic separation)
Readout probe 1	CTTGCGACGTCAGTGGA-TAGTGTCTTACACGATTT	5′ HRP (direct electrochemical readout)
Readout probe 2	CTTGCGACGTCAGTGGA-TAGTGTCTTACACGATTT	5′ DIG (indirect readout)
Readout probe 3	CTTGCGACGTCAGTGGA-TAGTGTCTTACACGATTT	5′ Cy3 (direct fluorescence readout)
Readout probe 4	AGAGTGTACCGACCTC	5′ Cy3 (direct fluorescence readout)

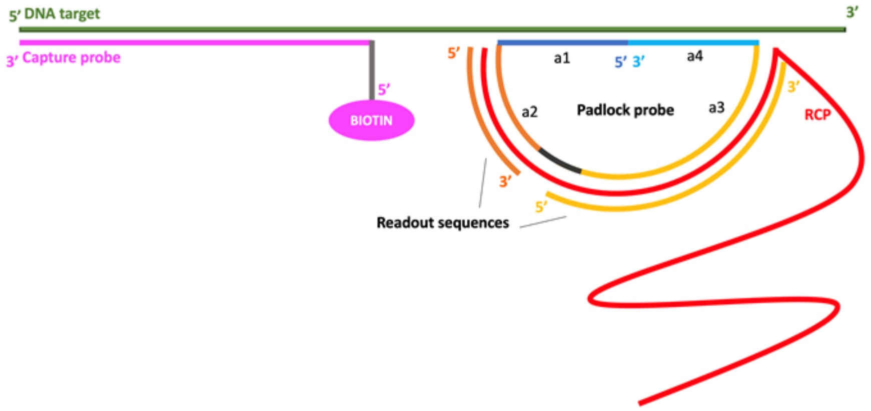

Figure S1. Design of the specific padlock probe for the detection of *E. coli* comprising two complementary sequences to the target (a1 and a4) and two sequences (a2 and a3) to achieve the readout. The schematic location of the capture probe is also Scheme 1. Figure A2, etc.

2.5. Rolling circle amplification on streptavidin magnetic particles and electrochemical genosensing

The procedure is schematically described in Figure 3. Each sample of DNA target was incubated for 20 min with 50 nmol L-1 biotinylated capture probe (Table 1), in order to achieve the preconcentration of the RCPs on streptavidin MPs. This reaction was performed in PLP ligation mix comprising 10 nmol L -1 PLP, 0.2 mg mL-1 BSA, 0.68 mmol L-1 ATP, 1x hybridization buffer and 5 U T4 ligase (Figure 3, panel A). Afterwards, 4 µL streptavidin-modified magnetic particles (streptavidin-MPs) were added to the ligation reaction mixture and incubated for 5 min at room temperature (RT) under rotation,

coupling the biotinylated target/PLP complex to the MPs via biotin-streptavidin bond (Figure 3, panel B). Under magnetic actuation, the supernatant was removed, and the particles were washed once in washing buffer. After that, RCA mix, containing 1x phi29 DNA polymerase buffer, 125 μM dNTPs, 0.2 mg ml -1 BSA, and 6 U phi29 DNA polymerase, was added to the particles and RCA was performed at 37°C for 60 min (Figure 3, panel C). The RCPs attached to the streptavidin-MPs were then hybridized with a readout probes labelled with HRP. In the direct labelling, this procedure was performed by the incubation with the readout probe 1 labelled with HRP (20 nmol L-1) in hybridization buffer A at 37 °C for 45 min (Figure 3, panel D), followed by washing (x3) to remove the unbound reagents. After each incubation or washing step, a magnetic separator was positioned under the tubes until pellet formation occurred on the tube's side wall, followed by supernatant separation. Finally, the measurement of RCPs attached on the streptavidin-MPs was performed on carbon screen-printed electrodes with a portable bipotentiostat connected by a universal USB port to a laptop computer operated by batteries. For this, each sample was resuspended in 60 μL of 0.25 mmol L^{-1} H_2O_2 and 1 mmol L^{-1} of hydroquinone (Figure 3, panel E, and Figure S4). After 2 min of reaction, the solution was added to the electrode surface and measured by SWV. The potential range was 0.1 to -0.7 V with potential step and amplitude of 10 mV and frequency of 1 Hz. The maximal signal obtained in the peaks was used for the electrochemical signal plotted in the results. Due to its high turn-over range, horseradish peroxidase enzyme increases the sensitivity of the method amplifying the electrochemical signal. HRP is oxidized catalyzing the reduction of hydrogen peroxide to water in the presence of hydroquinone. On the other hand, HRP is reduced again by oxidating hydroquinone to benzoquinone. Therefore, the final readout at the surface of the screen-printed electrode is based on the reduction of the benzoquinone (the oxidized form of HQ) by SWV.

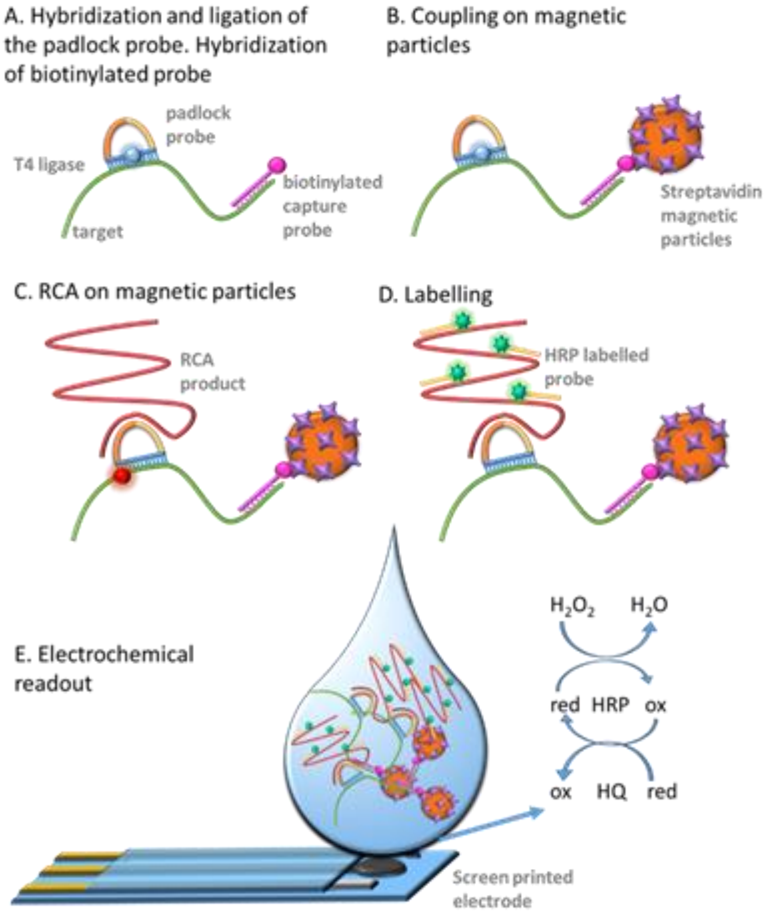

Figure S2. Schematic representation of the electrochemical genosensing for the rolling circle amplification products detection on streptavidin-MP. (A) The biotinylated capture probe as well as the padlock probe are both hybridized to the DNA target (B) The DNA target is then coupled on streptavidin magnetic particles; (C) RCA on the streptavidin-MP is then performed; (D) Hybridization with the readout probe (HRP labelled) to achieve the electrochemical reporter is then performed; (E) Electrochemical readout by square wave voltammetry upon addition of the mediator HQ and the substrate H_2O_2 for the HRP on screen-printed electrodes. **.**

Electrochemical readout

Carbon screen-printed electrodes were purchased from Dropsens (ref. DRP-110). These electrodes are printed using ceramics as a substrate and the electrochemical cell consists of a circular 4mm carbon working electrode, an auxiliary electrode made of carbon as well and silver as a reference electrode. The small dimensions of this electrodes (L 33 x W 10 x H 0.5 mm) make them ideal to work with the small volumes required in this work. The electrodes were connected to the boxed connector for SPE (Ref. DSC) which operates as an interface between the electrodes and the portable bipotentiostat (DRP-STAT200, DropSens) (Figure S3). The square wave voltammetry (SWV) measurements were performed in a laptop computer operated by the battery, in which the portable bipotentiostat was connected by a universal USB port.

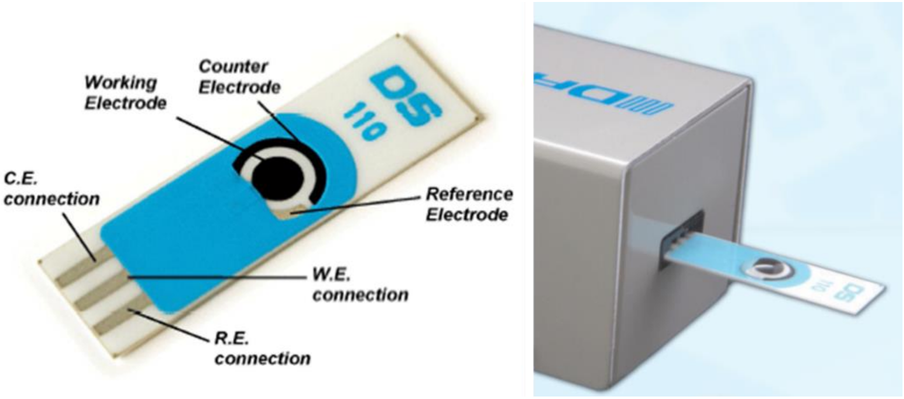

Figure S3. Configuration of the commercial screen-printed electrodes used in this work and image of the Boxed Connector for Screen-Printed Electrodes. It can be appreciated the drop on the Surface of the working carbon electrode (images obtained from Dropsens). .

The electrochemical readout was based on square wave voltammetry, in the presence of hydrogen peroxide (H_2O_2) as a substrate and hydroquinone (HQ) as mediator and the horseradish peroxidase enzyme (HRP) conjugated to the readout probes 1, which were used as electrochemical reporters. The sequences of these oligonucleotides are as follows:

Readout probe 1: HRP-CTTGCGACGTCAGTGGATAGTGTCTTACACGATTT

Readout probe 1 is complementary to the products of the rolling circle amplification (RCA).

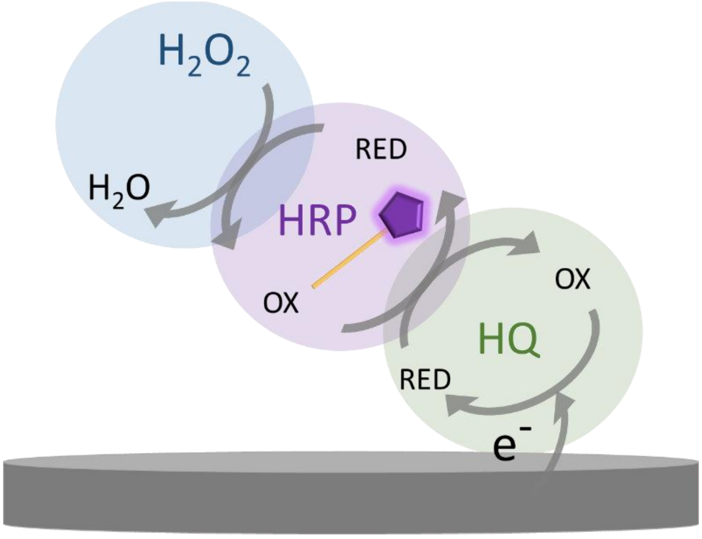

Figure S4. Enzymatic mechanism of the HRP enzyme conjugated to the readout probes on the surface of the carbon screen-printed electrode, upon addition of H_2O_2 as a substrate and hydroquinone (HQ) as a mediator.

Due to its high turnover range, horseradish peroxidase enzyme increases the sensitivity of the method amplifying the electrochemical signal. HRP is oxidized catalysing the reduction of hydrogen peroxide to water in the presence of hydroquinone. On the other hand, HRP is reduced again by oxidating hydroquinone to benzoquinone. Therefore, the final readout at the surface of the screen-printed electrode is based on the reduction of the benzoquinone (the oxidized form of HQ) by SWV.

For the square wave voltammetry measurements, 60 μL hydrogen peroxide 0.25 mmol L^{-1} and hydroquinone 1 mmol L^{-1} was added to the sample as substrate and mediator for the HRP enzyme conjugated to the readout probes (as shown in Figure 6.2, panel E). After 2 min of enzymatic reaction, the solution was transferred to the surface of the screen-printed electrode. The potential range used was from 0 to -0.7 V, with a potential step and amplitude of 10 mV, and frequency of 1 Hz. The data were recorded and processed using DropView 2.2 software. The resulting signal is directly proportional to the concentration of HRP, since the enzyme is working at saturation conditions. Hence the higher the hybridization with the readout HRP probe, the higher the cathodic signal.

Table S2. Analytical performance and main characteristics of other related biosensors or platforms based on isothermal amplification for the detection of *E. coli* or other bacteria.

Analyte	Detection system	Features	LOD	Ref
Escherichia coli	Electrical sensor	The amplified target are metalized with Au/Ag seeds producting a conductive metal wire	100 pM synthetic DNA. 66 fM genomic DNA	Russell, 2014 [1]
Salmonella	Fluorescence	RCPs product on beads are labeled with flourescent probes and visualised by microscopy	9 amol (equivalent to 9 pM)	Sato, 2013 [2]

Listeria monocytogenes	Electrochemiluminescence (ECL)	RCPs double-tagged with biotin for the immobilization on MPs, and tris(bipyridine) ruthenium for the eECL detection	10 aM synthetic DNA; 0.2 pg/ L genomic DNA	Long, 2011 [3]
Salmonella	Differential pulse voltammetry	AuNPs probes used for signal amplification combined with enzymatic reporter (alkaline phosphatase)	6.76 aM; 6 CFU/mL	Zhu, 2014 [4]
Synthetic DNA (no strain reported)	Differential pulse voltammetry	Use of molecular beacon for target immobilization on electrode surface. Alkaline phosphatase used as enzymatic reporter.	0.9 fM synthetic DNA	Cheng, 2014 [5]
E. coli	Square wave voltammetry	Amplification on magnetic particles combined with enzymatic reporter (peroxidase)	6.7 amol	This work

References

1. Russell, C.; Welch, K.; Jarvius, J.; Cai, Y.; Brucas, R.; Nikolajeff, F.; Svedlindh, P.; Nilsson, M. Gold nanowire based electrical DNA detection using rolling circle amplification. *ACS Nano* **2014**, *8*(2), 1147-53. Author 1, A.; Author 2, B. Title of the chapter. In *Book Title*, 2nd ed.; Editor 1, A., Editor 2, B., Eds.; Publisher: Publisher Location, Country, 2007; Volume 3, pp. 154–196.
2. Sato, K.; Ishii, R.; Sasaki, N.; Sato, K.; Nilsson, M. Bead-based padlock rolling circle amplification for single DNA molecule counting. *Anal Biochem.* **2013**, *437*(1), 43-5. Author 1, A.B.; Author 2, C. Title of Unpublished Work. *Abbreviated Journal Name* stage of publication (under review; accepted; in press).
3. Long, Y.; Zhou, X.; Xing, D. Sensitive and isothermal electrochemiluminescence gene-sensing of Listeria monocytogenes with hyperbranching rolling circle amplification technology. *Biosens Bioelectron.* **2011**, *26*(6), 2897-904.Author 1, A.B.; Author 2, C.D.; Author 3, E.F. Title of Presentation. In Title of the Collected Work (if available), Proceedings of the Name of the Conference, Location of Conference, Country, Date of Conference; Editor 1, Editor 2, Eds. (if available); Publisher: City, Country, Year (if available); Abstract Number (optional), Pagination (optional).
4. Zhu, D.; Yan, Y.; Lei, P.; Shen, B.; Cheng, W.; Ju, H.; Ding, S. A novel electrochemical sensing strategy for rapid and ultrasensitive detection of Salmonella by rolling circle amplification and DNA-AuNPs probe. *Anal Chim Acta.* **2014**, *846*, 44-50.
